# Inhibition of apoptosis signal-regulating kinase by paeoniflorin attenuates neuroinflammation and ameliorates neuropathic pain

**DOI:** 10.1186/s12974-019-1476-6

**Published:** 2019-04-11

**Authors:** Danli Zhou, Siqi Zhang, Liang Hu, Yu-Feng Gu, Yimei Cai, Deqin Wu, Wen-Tao Liu, Chun-Yi Jiang, Xiangqing Kong, Guang-Qin Zhang

**Affiliations:** 1 Department of Clinical Pharmacy, The Affiliated Wuxi People’s Hospital of Nanjing Medical University, Wuxi, 214023 China; 2 Department of Cardiology, The First Affiliated Hospital of Nanjing Medical University, Nanjing, 210029 China; 3 Neuroprotective Drug Discovery Key Laboratory of Nanjing Medical University, Department of Pharmacology, Nanjing Medical University, Nanjing, 211166 Jiangsu China; 4 Department of Pharmacy, The First Affiliated Hospital of Nanjing Medical University, Nanjing, 210029 China; 5 Department of Clinical Pharmacy, China Pharmaceutical University, Nanjing, 210009 China; 6 Neuroprotective Drug Discovery Key Laboratory of Nanjing Medical University, Department of Pharmacology, Nanjing Medical University, Nanjing, 210029 China

**Keywords:** Paeoniflorin, ASK1, Neuropathic pain, MAPK

## Abstract

**Background:**

Neuropathic pain is a serious clinical problem that needs to be solved urgently. ASK1 is an upstream protein of p38 and JNK which plays important roles in neuroinflammation during the induction and maintenance of chronic pain. Therefore, inhibition of ASK1 may be a novel therapeutic approach for neuropathic pain. Here, we aim to investigate the effects of paeoniflorin on ASK1 and neuropathic pain.

**Methods:**

The mechanical and thermal thresholds of rats were measured using the Von Frey test. Cell signaling was assayed using western blotting and immunohistochemistry.

**Results:**

Chronic constrictive injury (CCI) surgery successfully decreased the mechanical and thermal thresholds of rats and decreased the phosphorylation of ASK1 in the rat spinal cord. ASK1 inhibitor NQDI1 attenuated neuropathic pain and decreased the expression of p-p38 and p-JNK. Paeoniflorin mimicked ASK1 inhibitor NQDI1 and inhibited ASK1 phosphorylation. Paeoniflorin decreased the expression of p-p38 and p-JNK, delayed the progress of neuropathic pain, and attenuated neuropathic pain. Paeoniflorin reduced the response of astrocytes and microglia to injury, decreased the expression of IL-1β and TNF-α, and downregulated the expression of CGRP induced by CCI.

**Conclusions:**

Paeoniflorin is an effective drug for the treatment of neuropathic pain in rats via inhibiting the phosphorylation of ASK1, suggesting it may be effective in patients with neuropathic pain.

## Background

Neuropathic pain is a devastating chronic pain caused by primary lesions or diseases of the somatosensory nervous system [1]. Despite decades of extensive studies on its treatments and underlying mechanisms, the treatment of neuropathic pain continues to be a challenge for physicians [2]. Currently, nonsteroidal anti-inflammatory drug (NSAID), opioid, and their derivatives are standard medications for pain management [3]. Although these drugs are effective in treating acute pain, they fail to cure neuropathic pain [4]. Drugs currently under development mainly target ion channels in the peripheral nervous system. However, mounting evidence has indicated the crucial role of central sensitization in the pathophysiology of chronic pain [1, 5, 6]. Central sensitization represents augmented neuronal excitability and enhanced synaptic efficacy in nociceptive pathways in the spinal cord and the brain [7].

There are many ion channels and receptors on the cell membrane, involving in central sensitization [6, 8]. So, it is not realistic to suppress neuropathic pain by blocking only a single receptor or channel. Therefore, it is particularly important to find new analgesic targets, especially key molecules responsible for central sensitization. Convincing evidences show that the mitogen-activated protein kinase (MAPK) families are important for the central sensitization, especially chronic pain caused by glial cell-induced neuroinflammation during the induction and maintenance of chronic pain [9, 10]. The MAPK family has three major members including extracellular signal-regulated kinase (ERK), p38, and c-Jun N-terminal kinase (JNK) [11]. Nerve injury or spinal cord injury induces a profound activation of MAPKs in the spinal cord [12]. Interestingly, MAPKs reveal a cell-selective distribution. Increase in ERK activity is mainly distributed in neurons, while JNK activation is mainly reflected in astrocytes, and p38 activation is mainly reflected in microglia [13, 14]. Besides participating in pain, many articles also suggest that ERK is involved in nerve repair [15, 16]. However, the possibility of delaying repair may hinder the healing of neuropathic pain. Therefore, choosing the appropriate target molecule to inhibit the activation of JNK and p38 becomes a very appealing analgesic strategy.

Apoptosis signal-regulating kinase 1(ASK1) also known as mitogen-activated protein kinase kinase kinase 5 (MAP3K5) is a member of MAP kinase kinase kinase family and, as such, a part of MAPK pathway [17]. It activates JNK and p38, but having no effect on ERK, in a Raf-independent fashion in response to an array of stresses such as oxidative stress, endoplasmic reticulum stress, and TNF-α stimulation [18, 19]. It can be activated by multiple signals such as ROS, TNF-α, and TLR4 [20, 21]. Therefore, finding safe and effective small molecules to inhibit the activation of ASK1 may be a new effective way to treat neuropathic pain.

After screening a variety of compounds, we selected paeoniflorin, a biologically active molecule extracted from the root of *Paeonia lactiflora* pall, which has been used to treat chronic pain and arthritis in Japan and China for more than 1000 years [22, 23]. In this paper, molecular docking software was used to predict that paeoniflorin, which has been widely used in clinical trials in China, may have ASK1 inhibitory function. This study focused on the effect of paeoniflorin on Chronic constrictive injury (CCI)-induced ASK1 activity in the spinal dorsal horn and its analgesic mechanism. We hypothesized that paeoniflorin may inhibit the activation of ASK1 in the spinal dorsal horn and attenuate neuropathic pain in rats.

## Methods

### Animals and surgery

Adult male Sprague-Dawley rats (180–200 g) were provided by the Experimental Animal Center at Nanjing Medical University, Nanjing, China. The animals were housed five to six per cage under pathogen-free conditions with soft bedding under controlled temperature (22 ± 2 °C) and photoperiods (12:12-h light-dark cycle). They were allowed to acclimate to these conditions for at least 2 days before inclusion in experiments. For each group of experiments, the animals were matched by age and body weight.

CCI surgery was performed according to our previous study [24]. Rats were anesthetized with 4% pentobarbital sodium, and a 7-mm segment of the right common sciatic nerve was exposed at the mid-thigh level. Four ligatures (4-0 chromic catgut) thread at four sites with approximately 1-mm intervals were loosely tied around the nerve. The animals in the control group received identical surgery but without nerve injury.

### Drugs and reagents

NQDI-1 was purchased from Selleck Chemical Inc. (Houston, TX). Antibodies for p-p38 (Tyr182) (1:800, #9211S), ASK1 (1:1000, #8662S), pERK1/2 (Thr202/Tyr204) (1:1000, #4370), and p-JNK (Thr183/Tyr185) (1:1000, #9255S) and CGRP antibody (1:100, #14959) were purchased from Cell Signaling Technology (Beverly, MA). Antibody for GAPDH (1:5000, G9545) was purchased from Sigma-Aldrich Inc. (St. Louis, USA). Antibody for p-ASK1 (Thr845) (1:1000, bs-3031R) was purchased from Bioss (Woburn, MA). Antibodies for IBA-1 (1:100, ab178847), GFAP (1:100, ab7260), IL-1β (1:1000, ab200478), and TNF-α (1:1000, ab6671) were purchased from Abcam (Cambridge MA). Anti-mouse IgG, HRP-linked Antibody (1:3000, #7076) and Anti-rabbit IgG, HRP-linked Antibody (1:3000, #7074) were purchased from Cell Signaling Technology (Beverly, MA). All other chemicals were purchased from Sigma Chemical Co (St. Louis, MO).

### Assessment of CCI-related pain behaviors

Rats were performed according to our previous study [24]. The animals were placed in the testing environment daily for at least 2 days before baseline testing for acclimatization. Mechanical sensitivity was detected by Von Frey hairs (Woodland Hills, Los Angeles, CA) test. The animals were placed in boxes with elevated metal mesh floor for 30 min before testing. A series of Von Frey hairs with logarithmically incrementing stiffness were used to stimulate the plantar surface of each hind paw perpendicularly. Each rat was tested for three times, and the averages of the threshold were measured. For testing thermal hyperalgesia, rats’ foot withdrawal latency to heat stimulation was measured. An analgesia meter (UGO Basile, Italy) was used to provide a heat source. The animals were placed in boxes with a smooth and temperature-controlled glass floor. The heat source was focused on a portion of the hind paw, which was flushed toward the glass, so that a radiant thermal stimulus was delivered to that site. The stimulus shuts off when the hind paw withdrew (or the stimulus was removed after 20 s to prevent tissue damage). The intensity of the heat stimulus was maintained constant throughout all experiments. The elicited paw movement occurred at latency between 9 and 14 s in the control animals. Thermal stimuli were delivered three times to each hind paw at 5- to 6-min intervals. Behavioral tests were performed blindly.

### AutoDock

The three-dimensional (3D) structure of the Ask1 protein was retrieved from the RCSB Protein Data Bank database (http://www.rcsb.org/), and the PDB ID was 4BF2. Chemical structure of paeoniflorin (CAS NO. 23180-57-6) and the recognized Ask1 inhibitor NQDI-1 (CAS NO. 175026-96-7) were retrieved from PubChem database (https://pubchem.ncbi.nlm.nih.gov/), and the PubChem CID of the two ligands were 442534 and 5522952, respectively. PDBQT files of ligands (paeoniflorin and NQDI-1) and protein (Ask1) were prepared as described in the protocol [25]. Then, the protein-ligand docking studies were performed using the AutoDock Vina program [26], which is one of the widely used methods for protein-ligand docking. AutoDock Vina significantly improves the average accuracy of predictions. Here, PDBQT file of paeoniflorin and NQDI-1 was taken as ligand and PDBQT file of Ask1 was taken as protein. These ligands were docked with ASK1 around its important binding sites as described [27]. The prediction results and optimum ligand-bound conformations were performed with AutoDockTools [28, 29].

### Western blotting

To identify temporal expression or the phosphorylated levels of proteins, whole protein samples were analyzed. In brief, samples (cells or spinal cord segments at L1–L6) were collected and washed with ice-cold phosphate-buffered saline before being lysed in a radioimmunoprecipitation assay lysis buffer. Then, whole sample lysates were separated by SDS-PAGE and electrophoretically transferred onto polyvinylidene fluoride (PVDF) membranes (Millipore Corp., Bedford, MA). The membranes were blocked with 5% bovine serum albumin for 1 h at room temperature, probed with antibodies overnight at 4 °C with the primary antibodies, and then incubated with HRP-coupled secondary antibodies. Data were analyzed with the Molecular Imager (Gel DocTM XR, 170-8170) and the associated software Quantity One-4.6.5 (Bio-Rad Laboratories, Berkeley, CA).

### Immunohistochemistry

Under deep anesthesia, the animals were transcardially perfused with PBS followed by 4% paraformaldehyde, and L4 and/or L5 lumbar segment was dissected out and post-fixed in the same fixative. The embedded blocks were sectioned into 20-μm thick. The sections from each group (five mice in each group) were incubated with anti-CGRP antibody (1:100, #14959), anti-IBA-1 antibody (1:100, ab178847), and anti-GFAP antibody (1:100, ab7260). Then, the free-floating sections were washed with PBS and incubated with the secondary antibody (Alexa Fluor 488 AffiniPure Donkey Anti-Rabbit IgG, 1:300, #711-545-152, Jackson Laboratories, USA) for 2 h. After washing out three times with PBS, the samples were studied under a confocal microscope (Leica TCS SP2, Leica Biosystems, Wetzlar, Germany) for morphologic details of the immunofluorescence staining. Images were randomly coded and transferred to a computer for further analysis; the analyzed images were 8-bit grayscale.

### Statistical analyses

SPSS Rel 15 (SPSS Inc., Chicago, IL) was used to conduct all statistical analyses. Alteration of expressions of the proteins was detected, and the behavioral responses were tested with one-way ANOVA, followed by Bonferroni post hoc tests. Results were represented as mean ± SEM of the independent experiments. The mean fluorescent pixels of GFAP, IBA-1, and CGRP were measured by Image-Pro Plus 6.0. Results described as significant are based on a criterion of *P* < 0.05.

## Results

### CCI induced neuropathic pain and increased the expression of p-ASK1 in rats

**Fig. 1:**
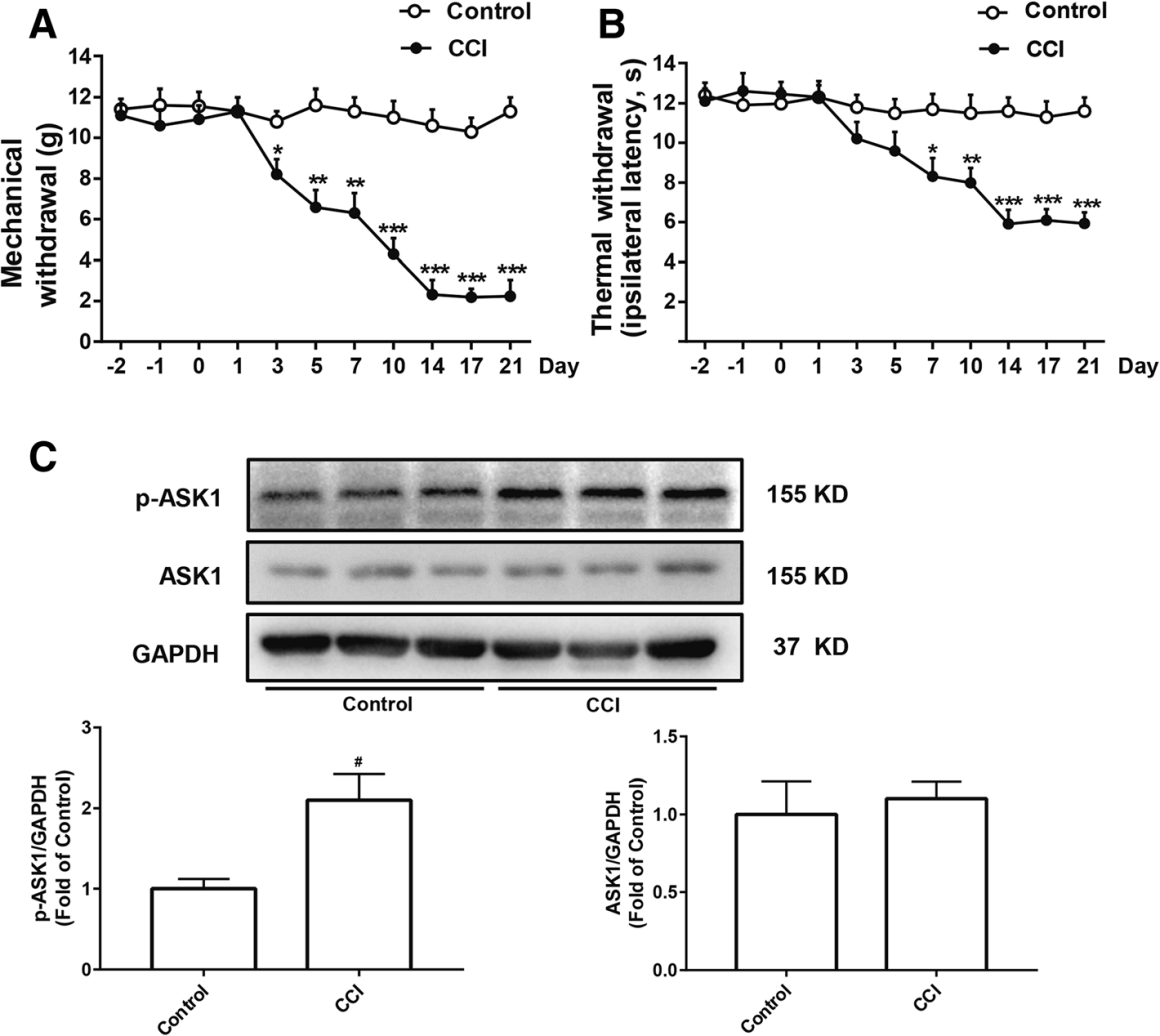
CCI induced neuropathic pain and increased the expression of p-ASK1 in the spinal cord of rats. **a**, **b** Mechanical allodynia and thermal hyperalgesia were significantly induced after CCI surgery (*n* = 8). **c** The expression of p-ASK1 and ASK1 were significantly increased at day 14 after CCI surgery in the spinal cord of rats. The lumbar spinal cords (L1–L6) were collected and analyzed 14 days after the CCI operation (*n* = 4). A significant difference was revealed following one-way ANOVA (*P < 0.05, **P < 0.01, ***P < 0.001 vs. control; Bonferroni post hoc tests)

Among several models of painful neuropathy have been developed in rats, CCI is the most commonly used neuropathic pain model to study the mechanism and to assess the effects of various treatments. As shown in Fig. 1a and b, the mechanical and thermal thresholds of rats, measured using the Von Frey test, were minimal at day 14 and last up to day 21 after CCI surgery. Mitogen-activated protein kinases (MAPKs: p38, JNK, and ERK MAPK) are critical for intracellular signal transduction and participate in regulating neural plasticity and inflammatory responses. Inhibition of MAPK pathways has been proved to attenuate inflammatory and neuropathic pain. ASK1, a member of the MAP3K family, could activate p38 MAPK and JNK pathway. To explore whether ASK1 play an important role in the process of neuropathy, we measured the phosphorylation of ASK1 using western blot. As shown in Fig. 1c, CCI increased the expression of p-ASK1 compared to the control group in the spinal cord of rats but did not affect the of ASK1 expression. These data suggested that CCI surgery could successfully decrease the mechanical and thermal thresholds of rats and the phosphorylation of ASK1.

### ASK1 inhibitor attenuated CCI-induced neuropathic pain and decreased the expression of p-JNK and p-p38

**Fig. 2:**
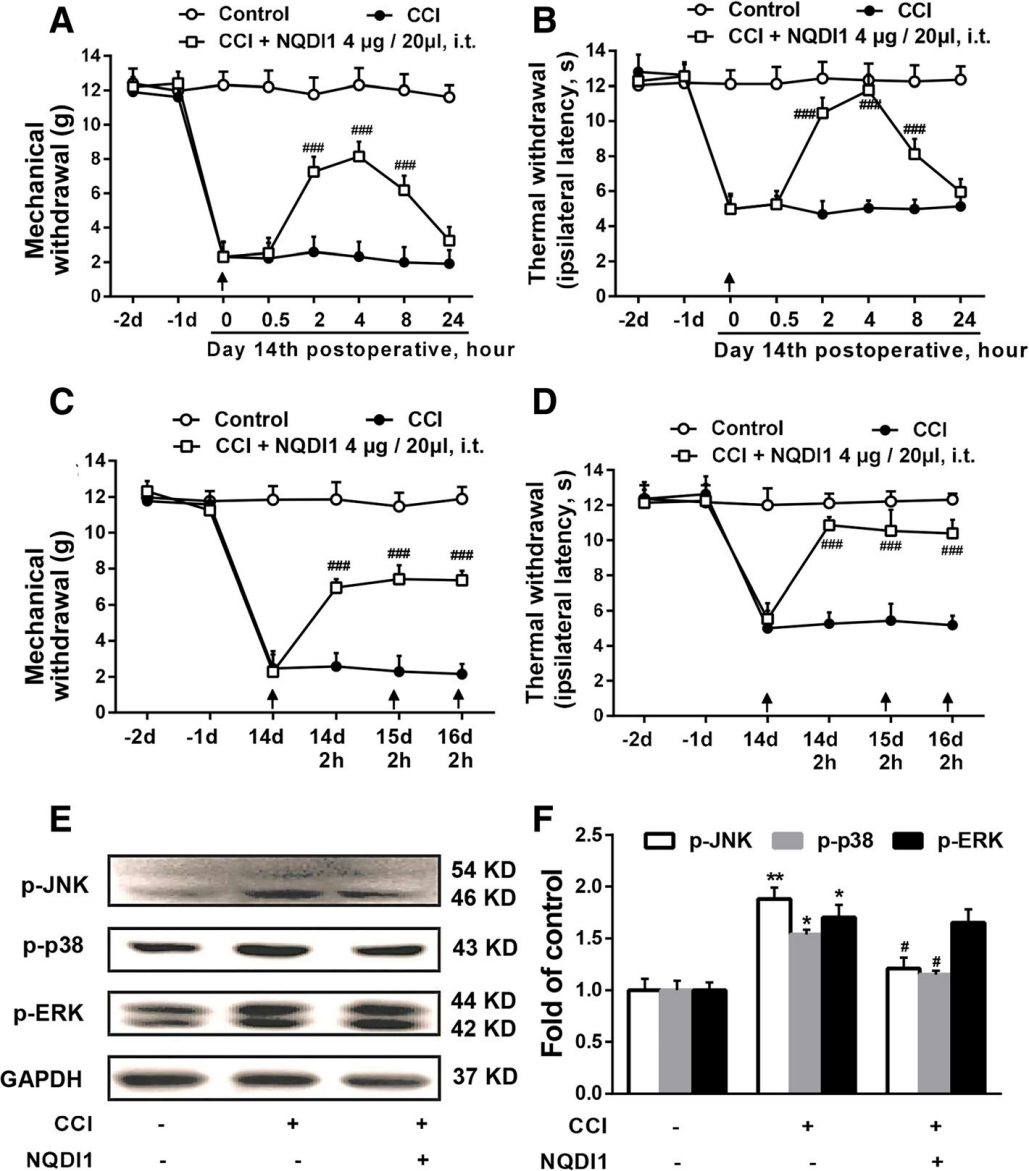
ASK1 inhibitor NQDI1 attenuated CCI-induced neuropathic pain and decreased the expression of p-p38 and p-JNK. **a**, **b** Single administration of ASK1 inhibitor NQDI1 (4 μg/20 μl, i.t.) at 14 days after CCI injury significantly attenuated CCI-induced mechanical allodynia and thermal hyperalgesia (*n* = 8). **c**, **d** Consecutive administration of ASK1 inhibitor NQDI1 (4 μg/20 μl, i.t.) at days 14, 15, and 16 after CCI injury significantly attenuated CCI-induced mechanical allodynia and thermal hyperalgesia (*n* = 8). **e**, **f** NQDI1 (4 μg/20 μl, i.t.) decreased the expression of p-JNK and p-p38 induced by CCI but did not affect p-ERK expression in the spinal cords of rats. The lumbar spines (L1–L6) were collected and analyzed 2 h after the drug administration (*n* = 4). A significant difference was revealed following one-way ANOVA (*P < 0.05, **P < 0.01, ***P < 0.001 vs. control; ^#^*P* < 0.05, ^##^*P* < 0.01, ^###^*P* < 0.001 vs. CCI group; Bonferroni post hoc tests)

Considering the significant changes of ASK1 in the neuropathic pain process, and lack of reports about the effect of ASK1 on the mechanical allodynia and thermal hyperalgesia, we measured the mechanical and thermal thresholds after ASK1 inhibitor NQDI1 administration. As shown in Fig. 2a and b, compared with the CCI group, ASK1 inhibitor NQDI1 (4 μg/20 μl, i.t.) significantly increased the mechanical and thermal thresholds in the ipsilateral paws of rats at 2, 4, and 8 h (*P* < 0.001) after a single administration of NQDI1. We then measured the analgesic effect of consecutive administration of NQDI1. As shown in Fig. 2c and d, ASK1 inhibitor NQDI1 (4 μg/20 μl, i.t. once daily for 3 days) markedly increased the mechanical and thermal thresholds in the ipsilateral paws of rats.

We further verified the effect of ASK1 inhibitor on MAPK kinases. As shown in Fig. 2e and f, compared with the control group, CCI increased the phosphorylation of p38, JNK, and ERK MAPK in the spinal cord of rats, and ASK1 inhibitor NQDI1 (4 μg/20 μl, i.t.) selectively alleviated the phosphorylation of p38 and JNK (*P* < 0.05), but did not affect the expression of p-ERK.

These data suggested that ASK1 inhibitor NQDI1 could attenuate neuropathic pain and decreased the phosphorylation of p38 and JNK.

### Paeoniflorin decreased the phosphorylation of ASK1 in vivo

**Fig. 3:**
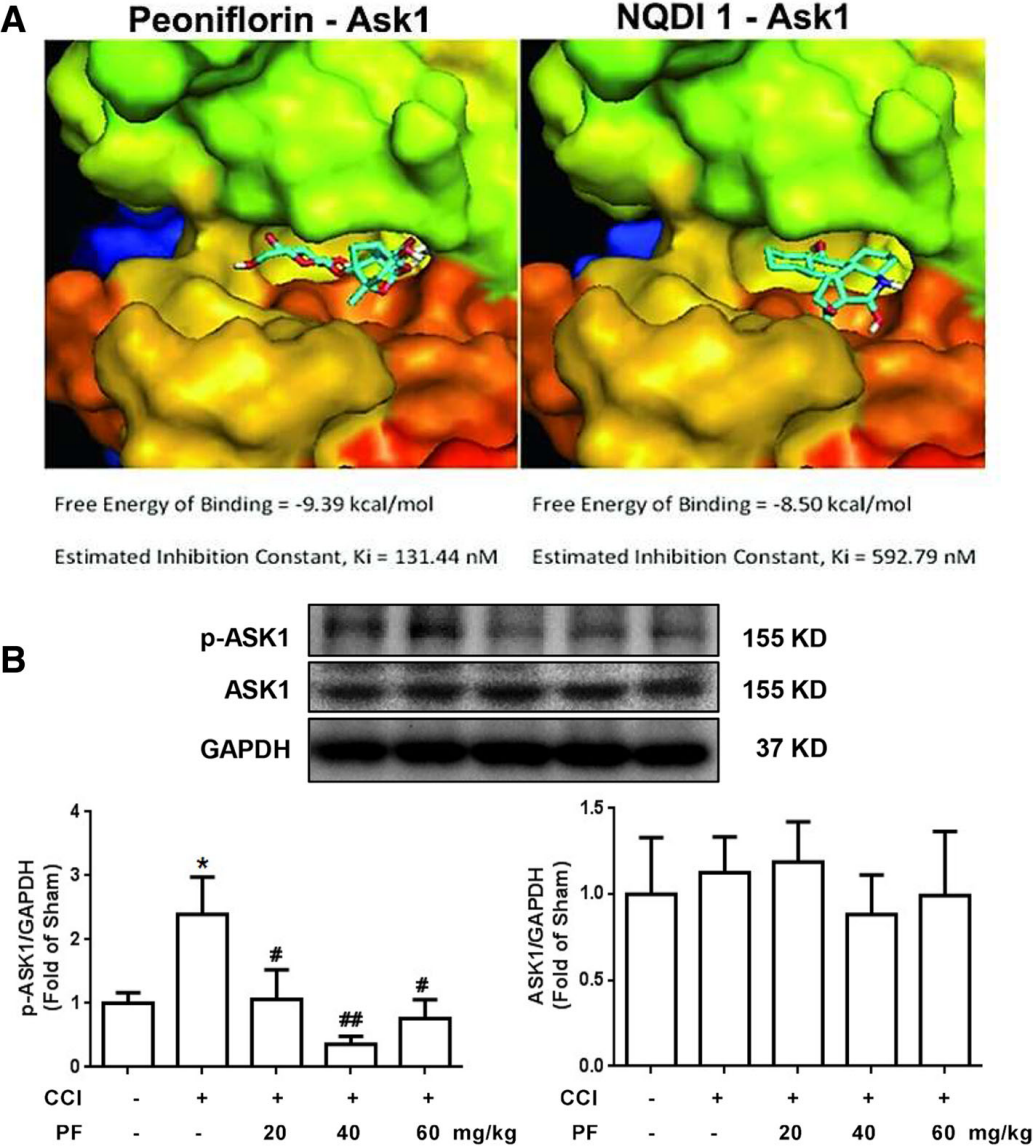
Paeoniflorin mimicked ASK1 inhibitor and decreased the phosphorylation of ASK1 in vivo. **a** AutoDock results show that paeoniflorin tightly bound to ASK1, which is equivalent to the ASK1 inhibitor NQDI1. **b** Paeoniflorin (20, 40, and 60 mg/kg, i.p.) significantly decreased the phosphorylation of ASK1 at 2 h after administration (*n* = 4). The lumbar spines (L1–L6) were collected and analyzed 2 h after the drug administration. PF, paeoniflorin. Significant differences were revealed following one-way ANOVA (*P < 0.05, **P < 0.01, ***P < 0.001 vs. control; ^#^*P* < 0.05, ^##^*P* < 0.01, ^###^*P* < 0.001 vs. CCI group; Bonferroni post hoc tests)

Currently, there is no effective medication for treating neuropathic pain via inhibiting ASK 1. Thus, we are trying to find drugs that are clinically used to inhibit ASK 1 phosphorylation. As shown in Fig. 3a, the results of AutoDock suggested that paeoniflorin, a monoterpene glucoside which is the principal bioactive component purified and extracted from the root of *Paeonia lactiflora* pall, tightly bound to ASK1, which is equivalent to the ASK1 inhibitor, NQDI1. We subsequently measured the effect of paeoniflorin on ASK1 phosphorylation. As shown in Fig. 3b, different doses of paeoniflorin (20, 40, and 60 mg/kg, i.p.) significantly decreased the phosphorylation of ASK1 at 2 h after administration. These data suggested that paeoniflorin may mimic the ASK1 inhibitor NQDI1 and inhibit ASK1 phosphorylation.

### Paeoniflorin prevented neuropathic pain-associated allodynia and hyperalgesia

**Fig. 4:**
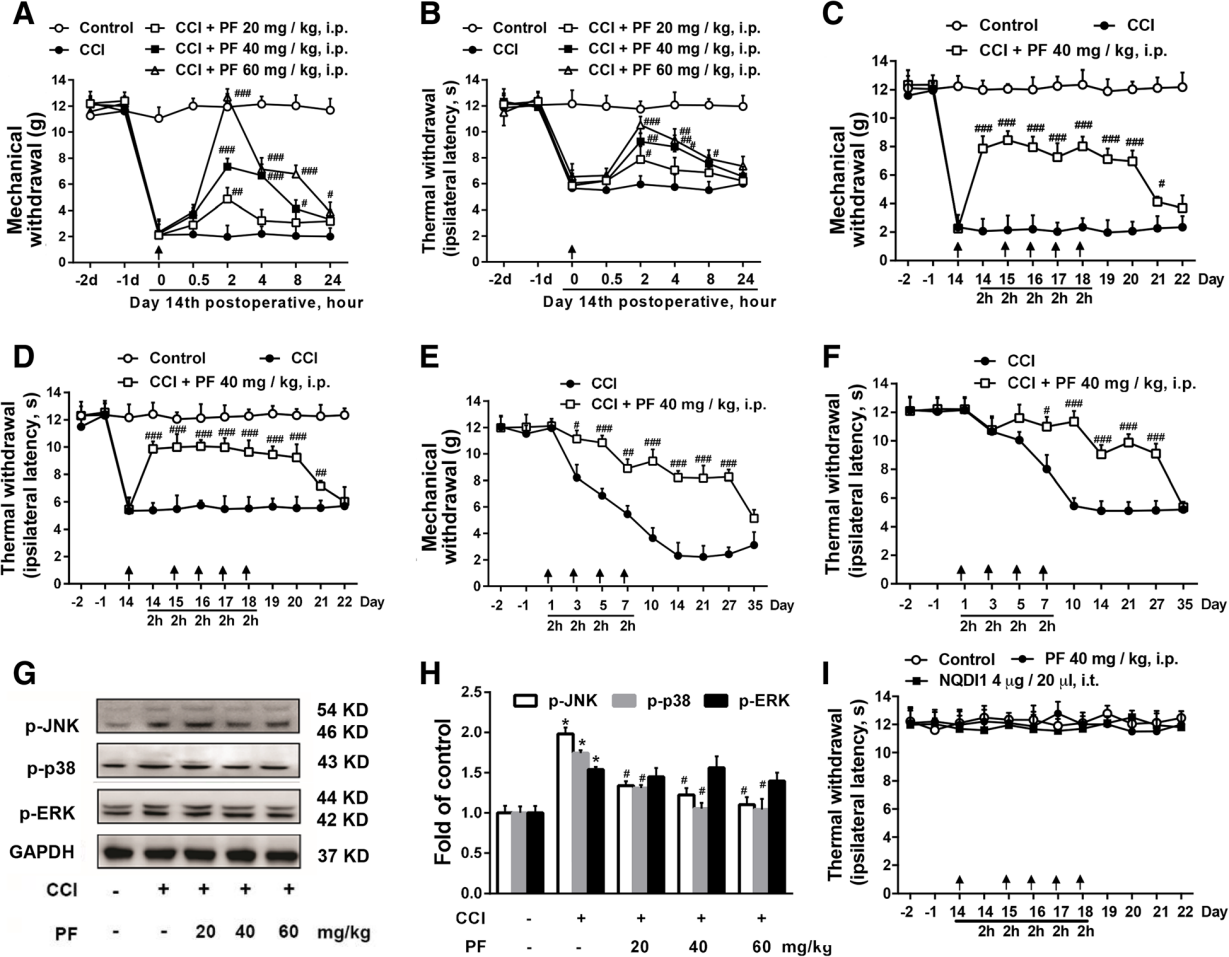
Paeoniflorin prevented neuropathic pain-associated allodynia and hyperalgesia. **a**, **b** Single administration of paeoniflorin (20, 40, and 60 mg/kg, i.p.) was administered at day 14 after CCI surgery, and paeoniflorin (40 and 60 mg/kg, i.p.) increased the mechanical and thermal thresholds in the ipsilateral paws of rats at 2, 4, and 8 h. Paeoniflorin (20 mg/kg, i.p.) increased the mechanical and thermal thresholds at 2 h (*n* = 8). **c**, **d** Consecutive administration of paeoniflorin (40 mg/kg, i.p.) significantly increased the mechanical and thermal thresholds in the ipsilateral paws up to day 21 after five times administration (*n* = 8). **e**, **f** Paeoniflorin (40 mg/kg, i.p.) significantly delay the development of neuropathic pain (*n* = 8). **g**, **h** Paeoniflorin (40 and 60 mg/kg, i.p.) decreased the phosphorylation of p38 and JNK but not ERK induced by CCI surgery (*n* = 4). **i** The effects of paeoniflorin and NQDI1 on mechanical and thermal thresholds in naive rats were measured. The lumbar spines (L1–L6) were collected and analyzed 2 h after the drug administration. PF, paeoniflorin. Significant differences were revealed following one-way ANOVA (*P < 0.05, **P < 0.01, ***P < 0.001 vs. control; ^#^*P* < 0.05, ^##^*P* < 0.01, ^###^*P* < 0.001 vs. CCI group; Bonferroni post hoc tests)

In consideration of the effect on ASK1 phosphorylation, we measured whether paeoniflorin could alleviate neuropathic pain like ASK1 inhibitor NQDI1. We measured the mechanical and thermal thresholds after paeoniflorin administration. As shown in Fig. 4a and b, compared to the CCI group, paeoniflorin (40 and 60 mg/kg, i.p.) increased the mechanical and thermal thresholds in the ipsilateral paws of rats at 2, 4, and 8 h after a single administration in a dose-dependent manner.

We then measured the analgesic effects of consecutive administration of paeoniflorin on 14 days after CCI surgery. As shown in Fig. 4c and d, paeoniflorin (40 mg/kg, i.p.) significantly increased the mechanical and thermal thresholds in the ipsilateral paws up to day 21 after five times of administration. We also found that paeoniflorin and NQDI1 have no effect on the mechanical and thermal thresholds in naive rats (Fig. 4i).

To explore the effect of paeoniflorin on the development of pain, paeoniflorin was administrated immediately after CCI surgery. As shown in Fig. 4e and f, paeoniflorin (40 mg/kg, i.p.) significantly delayed the development of neuropathic pain and the analgesic effect lasted until day 27.

Subsequently, we also measured the effects of paeoniflorin on MAPK kinases. As shown in Fig. 4g and h, paeoniflorin (40 and 60 mg/kg, i.p.) significantly decreased the phosphorylation of p38 and JNK induced by CCI surgery.

These data suggested that paeoniflorin could decrease the phosphorylation of p38 and JNK, delay the progress of neuropathic pain, and attenuate neuropathic pain, which is consistent with the experimental results of ASK1 inhibitor NQDI1.

### Paeoniflorin inhibited the response of astrocyte and microglia and reduced inflammation

**Fig. 5:**
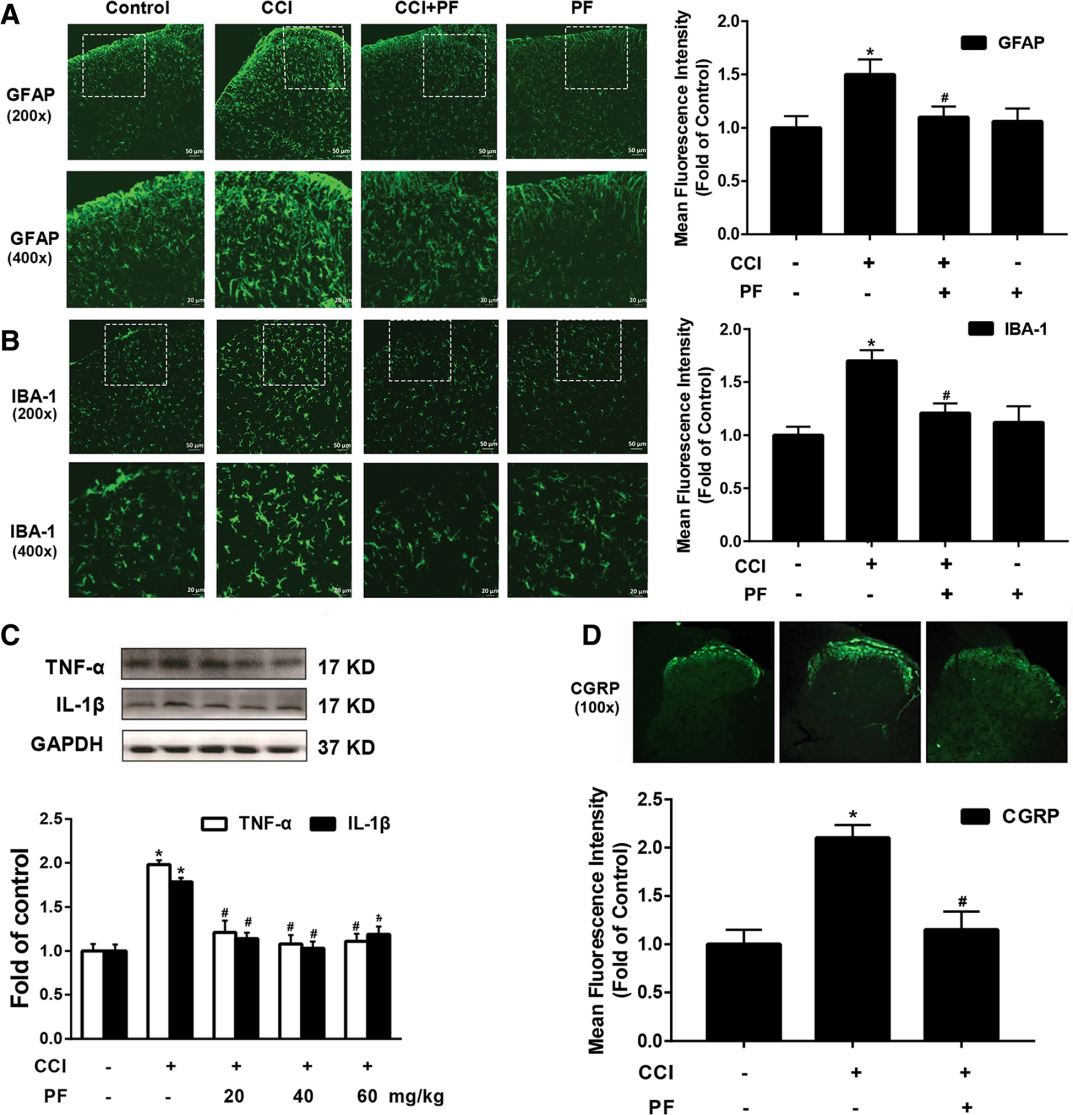
Paeoniflorin inhibited the response of astrocyte and microglia and reduced inflammation. **a**, **b** Paeoniflorin (40 mg/kg, i.p.) significantly decreased the expression of GFAP and IBA-1 induced by CCI in the spinal cords (*n* = 4). **c** Paeoniflorin (40 and 60 mg/kg, i.p.) significantly reduced the expression of IL-1β and TNF-α in the spinal cord induced by CCI in the spinal cords (*n* = 4). **d** Paeoniflorin administration for 5 days distinctly suppressed the upregulation of CGRP in the spinal cord (*n* = 4). The lumbar spines (L1–L6) were collected and analyzed 2 h after the last drug administration (day 18 after CCI surgery). Quantification of immunofluorescence was represented as mean fluorescence intensity in the superficial dorsal horns (*n* = 6 images per animal). PF, paeoniflorin. Significant differences were revealed following one-way ANOVA (*P < 0.05, **P < 0.01, ***P < 0.001 vs. control; ^#^*P* < 0.05, ^##^*P* < 0.01, ^###^*P* < 0.001 vs. CCI group; Bonferroni post hoc tests)

Microglia and astrocytes have recently emerged as key contributors to the pathological of neuropathic pain. To test the response of glial cells, GFAP and IBA-1 expression have been used as markers of astrocytes and microglia activity, respectively. As shown in Fig. 5a and b, compared with the CCI group, paeoniflorin (40 mg/kg) significantly decreased the expression of GFAP and IBA-1 induced by CCI. Chronic pain significantly induced the response of glial cells. Activated glia release a variety of neuroexcitatory substances that potentiate neurotransmission, especially proinflammatory cytokines (TNF-α and IL-1β). Compared with the CCI group, paeoniflorin (40 and 60 mg/kg, i.p.) significantly reduced the expression of IL-1β and TNF-α in the spinal cord induced by CCI (Fig. 5c).

Nitzan-Luques found the significance of calcitonin gene-related peptide (CGRP) in Aβ-touch afferents in the development of neuropathic pain. Intrathecal injection of CGRP could induce mechanical allodynia in naïve rats [30]. Moreover, administration of CGRP-P inhibitor almost reversed the allodynia in the model of spinal nerve ligation (SNL) [31]. Interestingly, we also found that paeoniflorin administration for 5 days distinctly suppressed the overexpression of CGRP in the spinal cord (Fig. 5d).

These data suggested that paeoniflorin could reduce the response of astrocytes and microglia, decrease the expression of IL-1β and TNF-α, and downregulate the expression of CGRP induced by CCI.

## Discussion

In this study, our major findings are as follows: (1) CCI significantly increased phosphorylation of ASK1 in the spinal cord of rats. (2) ASK1 inhibitor NQDI1 attenuated CCI-induced neuropathic pain. (3) Paeoniflorin decreased the phosphorylation of ASK1 and prevented neuropathic pain-associated allodynia and hyperalgesia. (4) Paeoniflorin inhibited the response of astrocytes and microglia and reduced inflammation.

ASK1 consists of 1379 amino acids in mice and 1375 amino acids in humans and contains a serine/threonine kinase domain in the middle region [32, 33]. The phosphorylation of threonine residues is critical for ASK1 activation [33, 34]. Given the highly regulated nature of ASK1, its activity is strictly controlled by various regulatory molecules. In normal conditions, ASK1 is a homooligomer, which binds to another ASK1 via its C-terminal coiled-coil domain [35]. The N-terminal coiled-coil domain of ASK1 binds to thioredoxin (Trx), which suppresses ASK1 kinase activity [36]. Various stimuli, such as oxidative stress, tumor necrosis factor-α (TNF-α), and Fas antigen activation, can phosphorylate serine/threonine kinase domain and activate ASK1 [37, 38].

Numerous studies have shown that activation of MAP kinases, particularly JNK and p38, contributes toward the pathology of neuropathic pain [5, 39]. Chronic constriction-induced sciatic nerve injury can induce significant activation of JNK and p38 in the spinal dorsal horn [40]. Importantly, administration of p38 inhibitor and JNK inhibitor ameliorates symptoms of neuropathic pain in rodent models [41, 42]. As the upstream of JNK and p38, previous studies have found that ASK1 inhibitors can significantly reduce the activation of JNK and p38 induced by strokes or spinal cord injury [43, 44]. Interestingly, the number of studies testing the role of ASK1 in chronic pain is limited. Therefore, we designed experiments to assess the role of ASK1 in CCI-induced neuropathic pain. We first examined the expression of p-ASK1 and ASK1 levels in rat spinal cord. As Fig. 1a and b show, CCI induces significant mechanical allodynia and thermal hyperalgesia in mice. We also demonstrated that CCI significantly induces the upregulation of p-ASK1, but not ASK1 expression in the lumbar spinal cord (Fig. 1c).

Since our results suggest that CCI can significantly induce ASK1 activation in the spinal cord, we then examined whether ASK1 activation has functional significance for CCI-induced neuropathic pain. Data show that either a single-dose or continuous administration of ASK1 inhibitor could significantly attenuate CCI-induced mechanical allodynia and thermal hyperalgesia (Fig. 2a–d). Moreover, ASK1 inhibitors could also significantly inhibit the upregulation of p-JNK and p-p38 level induced by CCI, but had no significant effects on the p-ERK level, which was consistent with the function of ASK1 as an upstream regulator of JNK and p38, but not ERK (Fig. 2e, f).

The role of ASK1 in the pathological process of pain is controversial, partly due to the lack of clinical methods to inhibit it. Thus, it is impossible to further investigate the analgesic effect of ASK1 inhibition. Computer simulations and molecular docking techniques were used to investigate various compounds. We found that paeoniflorin could efficiently dock with ASK1 (Fig. 3), and its dissociation constant was even lower than that of NQDI1, a classic ASK1 inhibitor. Therefore, we hypothesized that paeoniflorin may inhibit the activity of ASK1. Our results showed that paeoniflorin could significantly inhibit CCI-induced elevation of p-ASK1, but had no significant effects on ASK1 protein expression.

In accordance with the computer simulations and western blot results detailed above, behavioral tests also revealed that paeoniflorin attenuated CCI-induced neuropathic pain effectively (Fig. 4a–f). Our results indicated that paeoniflorin exhibited strong effects during the entire process of treatment regardless of its administration before or after the establishment of neuropathic pain. Paeoniflorin could also significantly inhibit the upregulation of p-JNK and p-p38 level induced by CCI, but had no significant effect on p-ERK level (Fig. 4g, h). These findings also supported our hypothesis that paeoniflorin alleviates neuropathic pain through the inhibition of ASK1.

Recently, many studies have found that ASK1 is expressed in glial cells and plays an important role in the induction and maintenance of neuroinflammation. Studies have found that inhibition of ASK1 can significantly inhibit the activation of JNK and p38, and significantly reduce inflammatory factors such as TNF and IL-1β released by glial cells, and even cytokines such as MMP-2/9 [43, 45, 46]. These inflammatory factors and cytokines are important mediators in the process of pain sensitization. Our results showed that paeoniflorin significantly inhibited the high expression of astrocyte marker GFAP and microglia marker IBA1 at the spinal cord level induced by CCI and significantly reduced the levels of TNF and IL-1β (Fig. 5a–c).

Calcitonin gene-related peptide (CGRP) is a peptide that is released by primary afferents and mediates the activation of NMDA receptors in neurons [47]. Calcitonin gene-related peptide upregulation is also believed to be a golden standard indicator of nociceptive activation [48]. Our immunofluorescence results indicate that paeoniflorin can significantly inhibit CCI-induced CGRP upregulation (Fig. 5d).

Considering that paeoniflorin was administered intraperitoneally in the present study, there is a possibility to induce other actions such as paeoniflorin induces SOCS3 expression and subsequently inhibits the TLR4 signaling pathway in the peripheral system, which has been proved in postoperative pain model in our previous study [49].

## Conclusions

In summary, paeoniflorin can not only significantly inhibit the activation of ASK1, simulate the analgesic effect of ASK1 inhibitors, but also significantly inhibit the response of glial cells and neuroinflammation induced by CCI. Given the importance of ASK1 in many neuroinflammation-related diseases and the excellent clinical safety history and low cost of paeoniflorin, our findings may represent a bright prospect for the development of ASK1 inhibitors based on paeoniflorin, but also provide a new perspective and basis for the development of novel analgesic and anti-inflammatory drugs based on paeoniflorin as a lead compound.

## Abbreviations

BSA: Bovine serum albumin
DMEM: Dulbecco’s modified Eagle’s medium
DMSO: Dimethyl sulfoxide
GAPDH: Glyceraldehyde-3-phosphate dehydrogenase
IL-1β: Interleukin-1β
MAPK: Mitogen-activated protein kinase
MMP-9/2: Matrix metalloproteinase-9/2
NMDA: *N*-methyl-d-aspartic acid
NR1: *N*-methyl-d-aspartate receptor 1
PBS: Phosphate-buffered saline
PC: Procyanidins
PKC: Protein kinase C
SDS-PAGE: Sodium dodecyl sulfate-polyacrylamide gel electrophoresis
